# Study protocol for a type III hybrid effectiveness-implementation trial of strategies to implement firearm safety promotion as a universal suicide prevention strategy in pediatric primary care

**DOI:** 10.1186/s13012-021-01154-8

**Published:** 2021-09-22

**Authors:** Rinad S. Beidas, Brian K. Ahmedani, Kristin A. Linn, Steven C. Marcus, Christina Johnson, Melissa Maye, Joslyn Westphal, Leslie Wright, Arne L. Beck, Alison M. Buttenheim, Matthew F. Daley, Molly Davis, Marisa E. Elias, Shari Jager-Hyman, Katelin Hoskins, Adina Lieberman, Bridget McArdle, Debra P. Ritzwoller, Dylan S. Small, Courtney Benjamin Wolk, Nathaniel J. Williams, Jennifer M. Boggs

**Affiliations:** 1 Perelman School of Medicine, University of Pennsylvania, Philadelphia, PA USA; 2 Center for Health Policy and Health Services Research, Henry Ford Health System, Detroit, MI USA; 3 School of Social Policy and Practice, University of Pennsylvania, Philadelphia, PA USA; 4 Institute for Health Research, Kaiser Permanente Colorado, Aurora, CO USA; 5 School of Nursing, University of Pennsylvania, Philadelphia, PA USA; 6 Department of Pediatrics, Henry Ford Health System, Detroit, MI USA; 7 Wharton School of Business, University of Pennsylvania, Philadelphia, PA USA; 8 School of Social Work, Boise State University, Boise, ID USA

**Keywords:** Pediatrics, Primary care, Behavioral economics, Evidence-based practice, Implementation science, Hybrid effectiveness-implementation trials, Violence prevention, Firearm safety promotion

## Abstract

**Background:**

Insights from behavioral economics, or how individuals’ decisions and behaviors are shaped by finite cognitive resources (e.g., time, attention) and mental heuristics, have been underutilized in efforts to increase the use of evidence-based practices in implementation science. Using the example of firearm safety promotion in pediatric primary care, which addresses an evidence-to-practice gap in universal suicide prevention, we aim to determine: is a less costly and more scalable behavioral economic-informed implementation strategy (i.e., “Nudge”) powerful enough to change clinician behavior or is a more intensive and expensive facilitation strategy needed to overcome implementation barriers?

**Methods:**

The Adolescent and child Suicide Prevention in Routine clinical Encounters (ASPIRE) hybrid type III effectiveness-implementation trial uses a longitudinal cluster randomized design. We will test the comparative effectiveness of two implementation strategies to support clinicians’ use of an evidence-based firearm safety practice, *S.A.F.E. Firearm*, in 32 pediatric practices across two health systems. All pediatric practices in the two health systems will receive *S.A.F.E. Firearm* materials, including training and cable locks. Half of the practices (*k* = 16) will be randomized to receive Nudge; the other half (*k* = 16) will be randomized to receive Nudge plus 1 year of facilitation to target additional practice and clinician implementation barriers (Nudge+). The primary implementation outcome is parent-reported clinician fidelity to the *S.A.F.E Firearm* program. Secondary implementation outcomes include reach and cost. To understand how the implementation strategies work, the primary mechanism to be tested is practice adaptive reserve, a self-report practice-level measure that includes relationship infrastructure, facilitative leadership, sense-making, teamwork, work environment, and culture of learning.

**Discussion:**

The ASPIRE trial will integrate implementation science and behavioral economic approaches to advance our understanding of methods for implementing evidence-based firearm safety promotion practices in pediatric primary care. The study answers a question at the heart of many practice change efforts: which strategies are sufficient to support change, and why? Results of the trial will offer valuable insights into how best to implement evidence-based practices that address sensitive health matters in pediatric primary care.

**Trial registration:**

ClinicalTrials.gov, NCT04844021. Registered 14 April 2021.

**Supplementary Information:**

The online version contains supplementary material available at 10.1186/s13012-021-01154-8.

Contributions to the literature

- Behavioral economics addresses how individuals’ decisions and behaviors are shaped by limited time and attention and insights can be incorporated into the development of low cost and scalable implementation strategies.
- Comparative effectiveness trials that determine whether behavioral economic-informed strategies can improve evidence-based practice use or if more intensive strategies are needed have significant implications for health policy and practice.
- The ASPIRE trial will test implementation strategies in two health systems in the USA to understand how best to implement firearm safety promotion in pediatric primary care to prevent youth suicide and unintentional injury.

## Background

Implementation science focuses on clinician behavior change within organizational constraints as a key target to improve care quality and patient outcomes [1]. A range of approaches from many disciplines, including organizational theory [2] and systems science [3] have been applied to understand how to change clinician behavior within organizations. One current limitation of the field is the assumption that clinicians maximize rationality and utility when making clinical decisions [4]. Behavioral economics focuses on how context and an individual’s limited resources (e.g., time, attention) shape decisions and behavior [5], and has identified common, predictable cognitive heuristics or shortcuts that people use in making decisions [6–8]. These heuristics can be harnessed through *choice architecture*, which involves changing the environment to facilitate the desired choice [9]. Implementation strategies informed by behavioral economics have been underused in efforts to increase the use of evidence-based practices. Deployment of these approaches through the electronic health record (EHR) can guide medical decision-making in ways that do not disrupt workflow and can be effective and low cost [10–13]. Given that more than 90% of hospitals, healthcare systems, and clinical practices in the United States (US) use an EHR [14], choice architecture strategies deployed in the EHR (e.g., a Best Practice Alert reminding clinicians to engage in evidence-based care)—hereafter called “Nudges”—are also highly scalable. EHR-delivered behavioral economic strategies have been used to change clinician practice in multiple areas of medicine and are highly promising [10, 15–18]. However, in the case of interventions targeting sensitive topics, such as firearm safety, sexual health behavior, or mental health and substance use, additional strategies may be needed to address clinician and practice factors such as clinician comfort with the intervention or leadership endorsement [19].

One promising strategy to address these barriers is implementation or practice facilitation (hereafter referred to as facilitation), an evidence-based implementation approach in which trained facilitators collaborate with local stakeholders to identify and address site-specific implementation barriers with the goal of building organizational capacity for improvement and increasing uptake and sustainment of the desired practice [20–22]. Although facilitation has been associated with increased clinician adoption of evidence-based practices and patient reach [23], it is resource-intensive, which may limit its scalability. Both scalability and effectiveness are key considerations when designing strategies to implement interventions addressing major public health problems, such as youth suicide by firearm. As such, in this trial, we will compare two approaches to implementing a firearm safety program in pediatric primary care as a universal suicide prevention strategy. Specifically, we will answer: is the less costly and scalable EHR-based “Nudge” powerful enough or is more intensive and expensive facilitation needed to overcome implementation barriers? We will also test the mechanisms through which our implementation strategies operate. We use firearm safety promotion as an example given the public health need [24], existing evidence-to-practice gap [25], and momentum nationally for health systems to play a role in reducing pediatric firearm injury and mortality [26].

### Research-to-practice gap: safe firearm storage program in pediatric primary care as a universal suicide prevention strategy

The US is experiencing a rise in youth suicide deaths. Firearms are the most common and lethal method of suicide attempt [27]. Reducing access to firearms is a promising yet underused suicide prevention strategy [28]. Addressing firearm storage is critical to suicide prevention efforts [29, 30] given that firearms are present in one in three US homes [31]. Recent research has found that seven in 10 firearm-owning families with children do not store all firearms in their home locked and unloaded as recommended by leading organizations including the American Academy of Pediatrics [32] and National Shooting Sports Foundation [33]. This means that approximately 4.6 million US children live in homes in which at least one firearm is stored unlocked and/or loaded [34]. Given that the presence of firearms in the home is a robust risk factor for suicide [35], safe storage of firearms in the home is imperative for reducing youth suicide attempts and death. Simulation research has found that even a modest increase in safer firearm storage could prevent as many as 32% of youth firearm deaths due to suicide and accidents [36]. Thus, efforts to increase implementation of interventions to improve secure firearm storage could save young lives nationally from suicide and unintentional injury.

### The evidence-based practice

*Safety Check* is an evidence-based pediatric primary care program targeting parental firearm storage as part of a bundle of violence prevention strategies that was originally developed for parents of youth ages 2–11 years [37]. The program, which is delivered by pediatricians and informed by a harm reduction approach aiming to reduce firearm injury, includes (1) screening for presence of firearms, firearm storage, and parental concerns about firearm injuries where children live and/or play; (2) counseling using brief motivational interviewing; and (3) providing firearm safe storage tools, such as cable locks, to parents. A large clinical trial found that parents receiving *Safety Check* reported double the odds of safe firearm storage (OR = 2.0, *p* < .001) compared to the control group. The intervention group showed a 10% increase in parent-reported use of cable locks, while there was a 12% decrease in cable locks in the control group. These results led major professional organizations to recommend use of *Safety Check*, but it has not been routinely implemented [38].

To increase our understanding of how best to implement *Safety Check* as a universal suicide prevention strategy [39], we conducted pre-implementation work in two health systems, guided by the Consolidated Framework for Implementation Research (CFIR) [40]. This work allowed us to gather key information about determinants (i.e., barriers and facilitators) to the implementation of *Safety Check* within the current national context, including clinician attitudes about discussing firearm safety with parents and the perspectives of firearm stakeholders (e.g., firearm safety course instructors) [38, 41]. This has paved the way for adaptations to *Safety Check* using an established adaptation framework [42, 43]. The adaptations made include expanding the reach to a broader age range (i.e., parents of children ages 5–17), changing the entry point of the counseling conversation from an identified parental concern to universal counseling for all parents, clarifying that firearm ownership status will not be documented in the EHR but that documentation may note that a conversation about firearm safe storage took place, offering additional resources from credible sources such as brochures or website links, and changing the program name. Based on crowdsourced feedback from parents, the program is now called *S.A.F.E. (Suicide and Accident Prevention through Family Education) Firearm* [43]. Our preliminary work also led to the development of implementation strategies using implementation mapping [44] to be tested in the proposed trial.

### Study contributions

The proposed research draws on multiple streams of evidence to maximize impact in the context of an urgent and sensitive topic and incorporates the latest advances in implementation science by merging behavioral economics and implementation science approaches. This offers an opportunity to test the support needed for implementation of *S.A.F.E. Firearm* and will also provide unique insights into implementation of sensitive evidence-based practices in primary care more broadly. Testing these implementation strategies in the context of a hybrid effectiveness-implementation trial may also reduce youth suicide and unintentional injury deaths. Additionally, despite the proliferation of conceptual frameworks [45, 46] and hypothesized determinants of practice within implementation science [47], little is known about which of the hypothesized determinants are causally related to implementation of evidence-based practices [1, 48] because very few trials test mechanisms or the processes responsible for change [49]. Our analysis of implementation strategy mechanisms will be critical to understanding how the strategies work and key to future efforts to optimize the effectiveness of our approaches. We will also gather information on associated implementation strategy costs to inform national scale-up efforts.

## Methods/design

This manuscript adheres to the Standards for Reporting Implementation Studies (StaRI) Statement (Additional file 1) [50].

The Adolescent and child Suicide Prevention in Routine clinical Encounters (ASPIRE) trial is a hybrid type III effectiveness-implementation trial [51] with a longitudinal cluster randomized design [52–54]. We will answer questions related to implementation strategy effectiveness in 32 pediatric and/or family medicine practices (henceforth referred to as “pediatric practices”) nested within two health systems within the Mental Health Research Network (MHRN), a National Institute of Mental Health-funded practice-based research network of 21 health systems. This study will be conducted in Henry Ford Health System (HFHS) and Kaiser Permanente Colorado (KPCO). During the active implementation period, 32 pediatric practices in the two health systems will receive *S.A.F.E. Firearm* materials, including brief training and cable locks. Half of the practices (*k* = 16) will be randomized to receive Nudge; the other half (*k* = 16) will be randomized to receive Nudge plus 1 year of facilitation to target additional clinician and practice implementation barriers (Nudge+). Trial study recruitment will start in 2022.

### Regulatory approvals

The ASPIRE trial was registered on ClinicalTrials.gov on April 14, 2021 (NCT04844021). The University of Pennsylvania institutional review board (IRB) serves as the single IRB (sIRB); reliance agreements were completed by both participating health systems. The study was approved on December 2, 2020 (#844327). The study is overseen by a data safety and monitoring board (DSMB) comprised of experts in implementation science methods, suicide prevention, and firearm safety promotion. The DSMB had an introductory meeting in February 2021 and will convene annually.

### Study team and governance

The study team includes an interdisciplinary group of researchers, clinicians, and health system stakeholders with expertise in implementation science, behavioral economics, firearm safety promotion, suicide prevention, biostatistics, mixed methods, and pediatric clinical care. The following consultants also contribute expertise to the study: the original developer of *Safety Check*, the developer of the hybrid design approach, and firearm safety experts (i.e., master firearm safety course instructors) who provide perspectives on the broader firearm landscape to ensure ecological validity of the work.

### Implementation framework, targets, and mechanisms

**Fig. 1:**
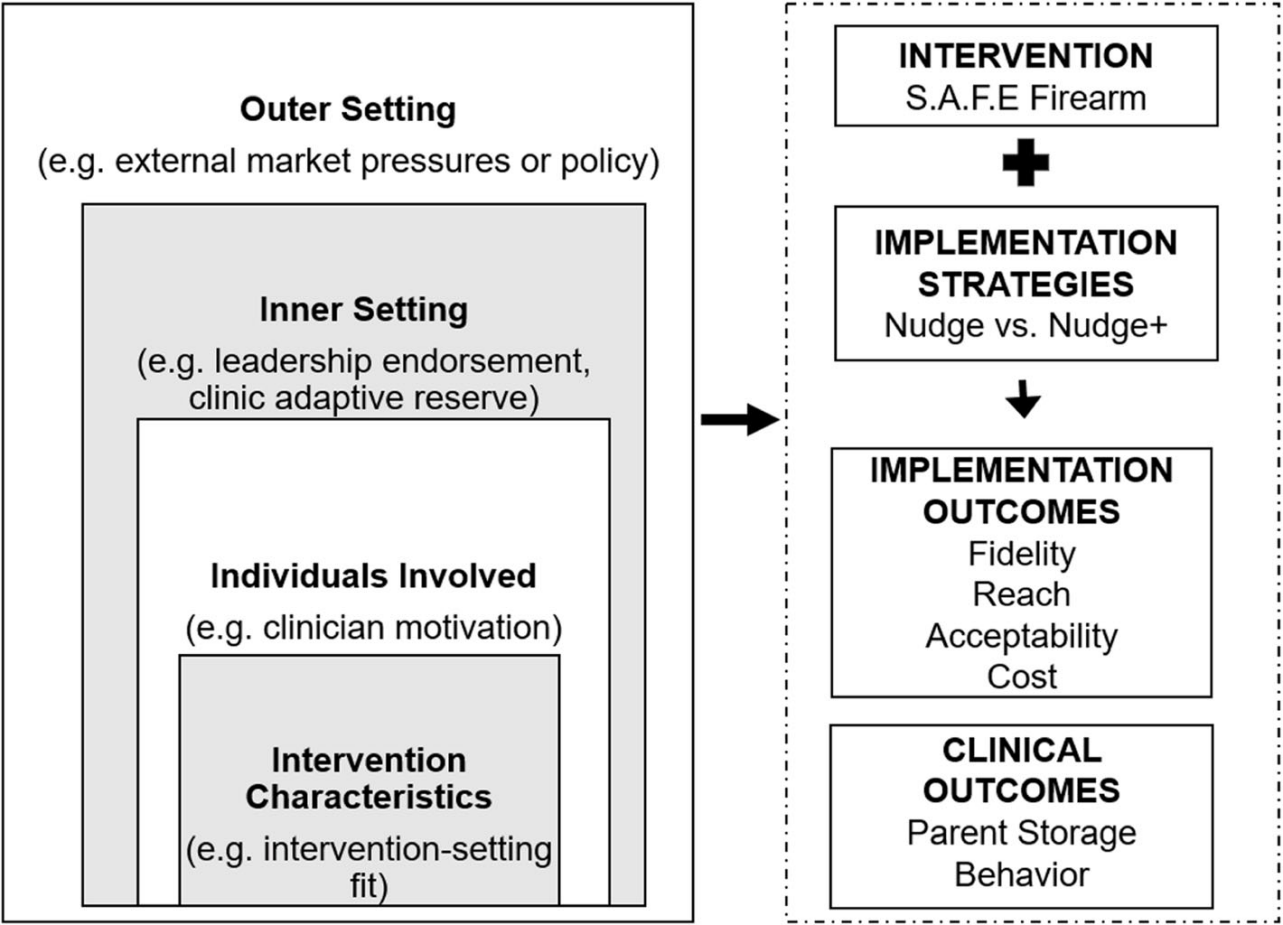
Guiding implementation frameworks. This figure depicts the contextual factors—guided by the Consolidated Framework for Implementation Research (CFIR) [40] (left)—that will be examined in relation to *S.A.F.E. Firearm* implementation and trial outcomes—guided by the Proctor et al. framework [55] (right)

Our research is guided by two implementation science frameworks: the Proctor et al. framework and CFIR [40, 55]. The Proctor et al. framework guides the relationship between our implementation strategies and implementation outcomes, listed in Fig. 1. Fidelity, operationalized as parent-reported clinician delivery of the two components of *S.A.F.E. Firearm* (brief counseling around firearm safe storage, offering cable locks), is the primary study outcome. Secondary outcomes include reach (EHR-documented program delivery) and acceptability (i.e., parent- and clinician-report of acceptability via online survey) of *S.A.F.E. Firearm* as well as implementation strategy costs [55]. CFIR guides our understanding of mechanisms related to inner setting factors (i.e., clinician and practice factors) that may mediate and/or moderate the relationship between implementation strategies and fidelity. Our primary mechanism of interest is practice adaptive reserve, a self-report practice-level measure composed of six factors: infrastructure, facilitative leadership, sense-making, teamwork, work environment, and culture of learning.

### Study aims and approach

#### Setting

**Fig. 2:**
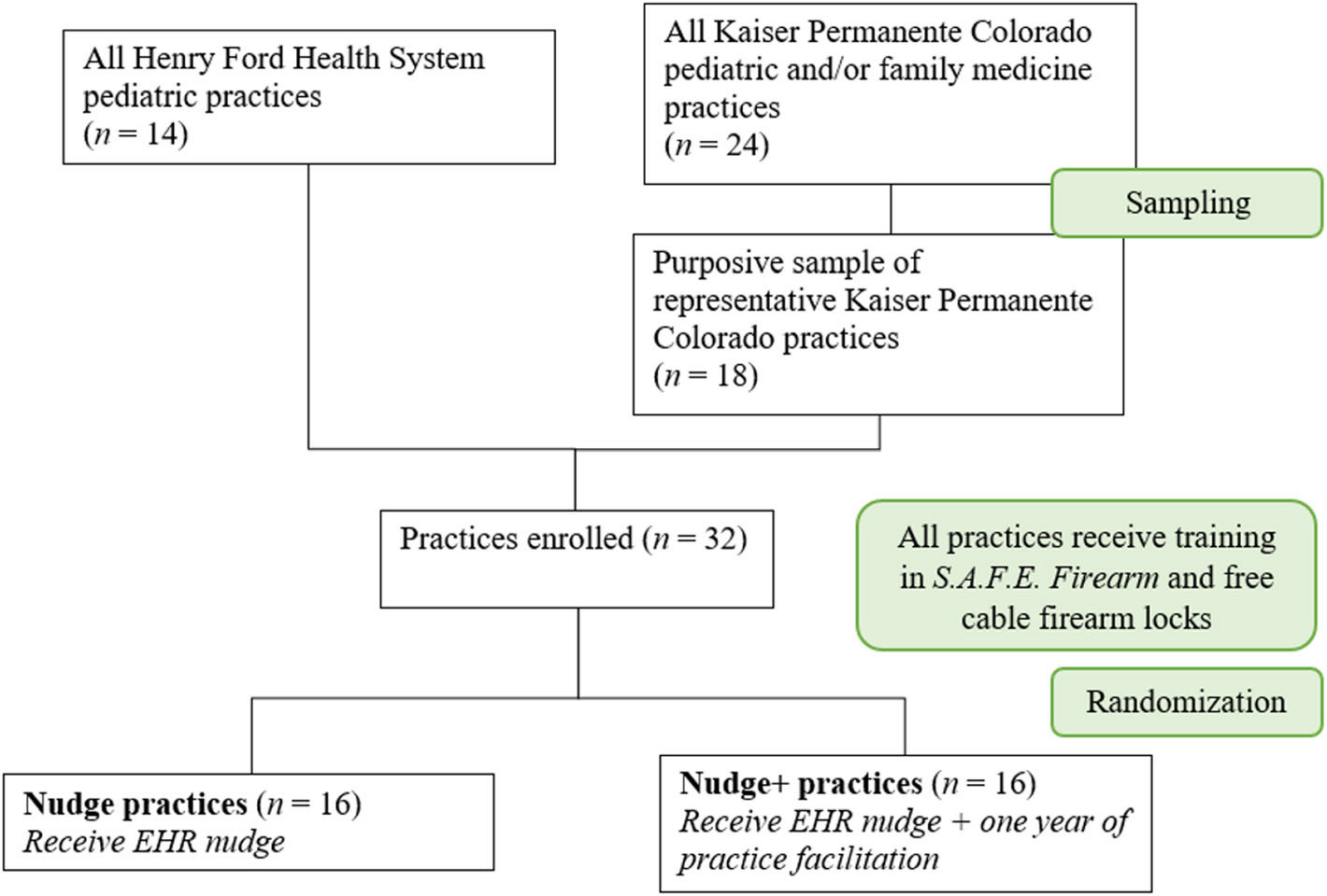
CONSORT diagram

We will conduct the proposed study in two geographically diverse MHRN systems that serve urban, suburban, and rural communities to maximize generalizability of our findings. HFHS (Michigan) includes the Detroit metro area and serves over 1.25 million patients per year, 38% of whom are racial or ethnic minorities. This is important given evidence of racial and ethnic disparities in suicide generally [56, 57] and firearm injury and mortality specifically [57, 58]. HFHS includes seven hospitals and more than 50 ambulatory care practices, 14 of which are pediatric practices. Our second partner, KPCO, serves approximately 600,000 members across Colorado including urban, suburban, and rural samples. It has 27 ambulatory care practices, including 24 pediatric practices (some stand alone, some are multi-specialty clinics), of which we will purposively choose 18 representative practices to participate. Thus, we will include 32 practices across the two sites. (Please see Fig. 2, CONSORT diagram.) Both health systems use the Epic electronic health record system. Recent estimates indicate that 45% of households in Colorado and 40% of households in Michigan owned firearms [59], putting Colorado above the national average of ownership [31].

#### Participants

Participants will include parents of youth seen in pediatric primary care, pediatric and family medicine clinicians (hereafter referred to as “clinicians”), and health system leaders. Clinicians delivering the *S.A.F.E. Firearm* program will include physicians (MD, DO) and advanced practice clinicians (nurse practitioner, physician assistant) who regularly conduct well-child visits with children and work in pediatric or family medicine departments.

##### Parents of youth seen in pediatric primary care

We will include parents and/or legal guardians (hereafter referred to as “parents”) at participating pediatric practices who have a child ages 5–17 years who attends a well-child visit. At least one parent must attend to be eligible. Our target age range of youth reflects the fact that suicide is the second leading cause of death among youth ages 10 and over [60], and rates are increasing in youth ages 5–12, particularly among Black or African American children [61–63]. Our upper limit is based on the age when most young people transition out of pediatrics in the participating health care systems. To optimize ecological validity, there are no exclusion criteria. We expect an *N* of approximately 58,866 eligible youth over the course of one year.

##### Clinicians and health system leaders

There are currently 137 physicians and 14 non-physician clinicians within the two systems who see young people within pediatrics and family medicine. Leaders (*n* = 20) include practice and department chiefs and health plan directors.

#### Evidence-based practice/intervention

**Fig. 3:**
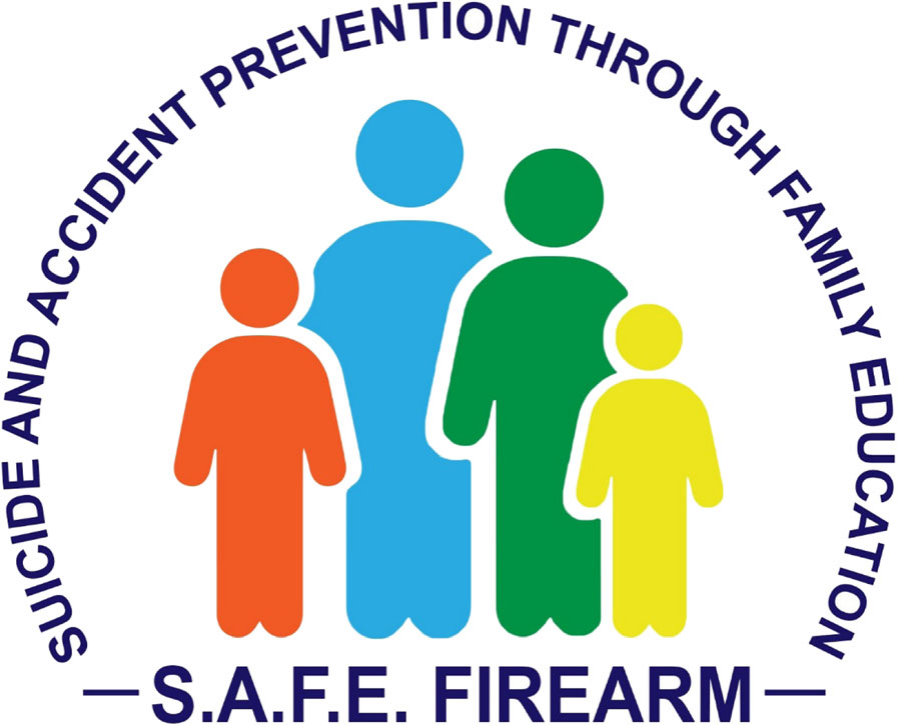
S.A.F.E Firearm name and logo based on crowdsourcing. *S.A.F.E. Firearm* name and logo, which was identified based on feedback from firearm owning and non-firearm owning parents [43]

*Safety Check* was developed using social cognitive theory [64] and uses a harm reduction approach to meet parents where they are with regard to their storage behavior [65, 66]. For this study, we will deploy an adapted version of *Safety Check* which maintains the key components of the original intervention (i.e., counseling and offering a cable lock) [37, 67] but extends its reach and acceptability [19, 38, 41]. Drawing on the ADAPT-ITT framework [42], we collaborated with parents, firearm safety experts, clinicians, and health system leaders [19, 38, 41, 43] to adapt *Safety Check* to reach a broader age group (i.e., youth < 18) and to serve as a universal suicide prevention strategy in pediatric primary care. Parents have been involved in the selection of name and logo (see Fig. 3); the program is now renamed *S.A.F.E Firearm*. Both firearm-owning and non-firearm-owning parents reported high acceptability of the adapted program [68, 69].

#### Implementation strategies

Prior to randomization, all 32 practices will receive *S.A.F.E. Firearm* materials and training. Clinicians will be strongly encouraged by pediatric leadership to access brief online training prior to trial launch [70, 71]. The video will include targeted information on how to counsel parents about firearm safety using motivational interviewing, an evidence-based approach that takes a nonjudgmental stance.

##### Nudge

All participating practices will receive the Nudge, which will be delivered via the EHR. During the study’s preparation phase, we will work with pediatric practice leadership and Epic information technology specialists to refine the design and functioning of our Nudge. We will prototype and pilot the Nudge to ensure it is consistent with current workflow, effective, and unobtrusive. We have decided to use a EHR SmartList, which is a pre-defined list of choices that users can select using their mouse or keyboard and are particularly helpful for documenting values that a clinician is required to use repeatedly, thus saving time and keyboard strokes. SmartLists are already used for other types of visit documentation in both health systems, which means clinicians are familiar with their functionality. We will add a default SmartList to the standard “Well-Child Visit” documentation template to serve as a Nudge and allow for tracking of *S.A.F.E. Firearm* implementation. The clinician will be asked to select a value from a drop-down list (e.g., “Discussed safe firearm storage” or “Did not discuss safe firearm storage;” “Offered a cable lock” or “Did not offer a cable lock”). Clinicians will be trained in how to document intervention delivery as part of annual training requirements. Our Nudge condition is informed by behavioral economic theory by enabling choice and bringing the desired behavior to the attention of the clinician [72]. While a hard stop in the EHR requiring a decision or a default where the desired behavior is preselected is likely more powerful [18], our approach is responsive to health system stakeholder preferences.

##### Nudge+

Practices randomized to this condition will receive the Nudge as described above, as well as 12 months of facilitation [73]. The role of the facilitator is to engage with study practices, to assist each practice in setting change and performance goals around the implementation of *S.A.F.E. Firearm*, and to troubleshoot implementation barriers.

Our approach to facilitation is informed by established facilitation manuals (i.e., Veteran Health Affairs Quality Enhancement Research Initiative [QUERI] facilitation manual [21, 74] and Agency for Healthcare Research and Quality [AHRQ] practice facilitation manual [22]) and includes six stages. First, facilitators will engage in an informal pre-implementation readiness assessment with each practice to identify potential implementation barriers and to develop relationships with stakeholders. Second, facilitators will support practices in addressing these barriers and launching the implementation strategy activities. These activities include identifying where in the workflow *S.A.F.E. Firearm* can be implemented, when *S.A.F.E. Firearm* will be delivered during the well-child visit, who in the practice will be responsible for storing the cable locks, where the locks will be stored, and other workflow matters. In keeping with behavioral economic principles, we will pay close attention to cable lock storage locations so locks can serve as visual reminders of the program (e.g., in baskets by documentation stations). Third, in the first 3 months of the active implementation period, facilitators will work with practices to set goals and establish metrics to monitor *S.A.F.E. Firearm* implementation. During this period, the facilitator will regularly engage with practice leadership and clinicians. In addition, facilitators will begin to develop a sustainment plan in collaboration with stakeholders. Fourth, in months 3–9, the facilitators will continue to work with practices to address barriers identified in the pre-implementation phase as well as new barriers that emerge as clinicians and practices begin implementing. This includes established implementation strategies such as Plan-Do-Study-Act cycles [75] and audit and feedback [76]. Fifth, in months 9–12, facilitators will engage in continued efforts to maintain gains and begin to enact the sustainment plan in preparation for the end of facilitation. Sixth, in month 12, facilitation activities will end, and the practices will transition to the formal sustainment period. Over the course of the active implementation period, facilitators (i.e., members of the study team who are trained in facilitation and include masters and doctoral level prepared colleagues) will offer expert consultation (i.e., webinars and technical assistance via email and phone as needed) and regular peer-to-peer calls supported by facilitators where practices can share their experience. All activities will be tracked via logs [21, 74] to ensure the ability to measure which strategies are delivered via facilitation (i.e., implementation fidelity).

#### Randomization

We will randomize practices to the active implementation conditions (Nudge [*k* = 16] or Nudge+ [*k* = 16]), using covariate-constrained randomization [77]. Covariate-constrained randomization enumerates a large number of possible assignments of the strategies to the practices and quantifies the balance across arms with respect to a set of pre-specified covariates for each one. Then, from a subset of possible assignments that achieve adequate balance, one is randomly chosen as the final allocation of strategies for the study. We will implement this randomization procedure to achieve balance with respect to three practice-level covariates: health system, practice size, and percent of patient panel that lives in a rural (i.e., non-metropolitan) [78] area based on geocoded patient home address.

#### Study timeline

**Fig. 4:**
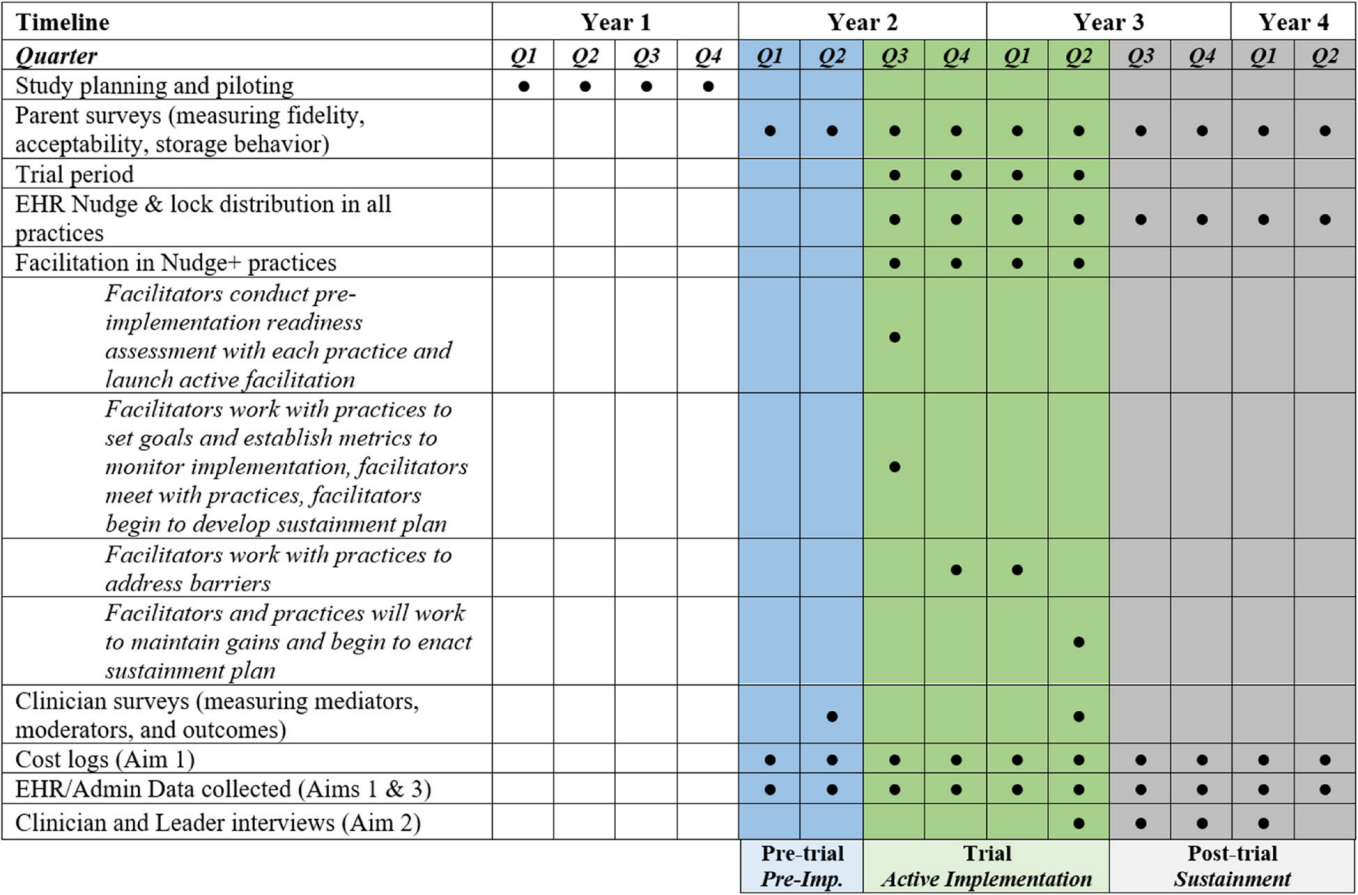
Study timeline. Timeline depicting study phases (pre-trial “pre-implementation” phase, trial “active implementation” phase, and post-trial “sustainment” phase) and study activities

Year 1 will be devoted to carefully planning and piloting our procedures to optimize our approach, including the collection of our primary outcome. In Year 2, we will begin collecting parent-reported clinician fidelity to allow us to capture baseline rates. The trial will launch in Year 2 and run for 12 months. During this period, both systems will deploy the EHR Nudge in all practices. Practices randomized to the Nudge+ condition will also receive facilitation. In years 3 and 4, the Nudge will continue in all practices but facilitation will be discontinued in the Nudge+ practices; we will continue to collect data from all practices to look at sustainment for 1 year. We will collect survey, interview, practice logs, and EHR data to answer study questions and test hypotheses. Aim 1 will examine the effects of Nudge+ relative to Nudge on parent-reported clinician fidelity, reach, cable lock distribution, acceptability, and implementation cost [55, 79]. See Fig. 4.

#### Aim 1: Examine the effects of Nudge vs. Nudge+ on implementation outcomes

##### Primary outcome

Fidelity is defined as a patient-level outcome indexing whether the patient received *S.A.F.E. Firearm* as prescribed by the program model; we call this “target *S.A.F.E. Firearm*.” The achievement of this outcome requires the patient’s clinician to follow both intervention steps (i.e., counseling and offering cable locks). Patients’ receipt of target *S.A.F.E. Firearm* will be measured via the following yes/no questions on a parent survey: (a) did someone on the healthcare team talk to you about firearm storage during your child’s recent visit? and (b) were free cable firearm locks made available to you during your child’s recent visit? Patients will receive a binary fidelity score indicating whether the clinician completed both (a) and (b) with them. In addition, we will code whether the steps occurred separately for supplemental analyses.

##### Secondary outcomes

Reach, or the number of parent-youth dyads who receive the intervention divided by the number of eligible parent-youth dyads [79], will be extracted from EHR data, based on clinician responses to EHR documentation. EHR data collection represents an exceptional opportunity to understand clinician behavior with all parents of youth rather than restricting data collection to a subset of clinicians who self-select to provide self-report or allow observation of their behavior [80], and we will be able to determine the entire clinical population denominator rather than the sample denominator.

As an additional measure of reach, the number of cable locks distributed in each practice will be recorded by research staff on a monthly basis. Because families will be permitted to take more than one lock, this metric will offer a proxy for the maximum number of firearms that may have been secured due to the intervention.

Acceptability will be measured from the perspective of both parents and clinicians. The parent survey will inquire about the acceptability of each *S.A.F.E. Firearm* program component separately with a single yes/no item (i.e., I found/would have found it acceptable to talk about firearm storage during my child’s visit; I found/would have found it acceptable to have free cable firearm locks made available to me during my child’s visit). Clinicians will rate the acceptability of each *S.A.F.E. Firearm* program component and implementation strategy separately via a single item rated on a six-point Likert scale (*strongly disagree* to *strongly agree*). This approach is based on our previous work assessing clinician acceptability of firearm safety programming [38].

To collect fidelity and acceptability data, all eligible parents in both health systems will be contacted within 2 weeks of their completed well-child visit, via email, mail, patient portal message, text message, or phone call by research specialists employed by their respective health system. The message will invite them to complete a survey via REDCap, a secure, web-based application for collecting and managing survey data that can be completed via computer or mobile device [81]. Follow-up contacts (e.g., phone calls, texts) will be made up to approximately 4 weeks after the well-child visit to enhance response rates. Follow-up recruitment strategies will differ and will be informed by best practices at each respective health system. Participants will be eligible for an incentive via lottery for survey completion (e.g., $100 gift card). We anticipate that we will be able to obtain responses from approximately 18,665 individuals using these methods.

To collect acceptability data, clinicians (*N* = 151) will be contacted via email using the Dillman Tailored Design Method [82] to boost response rates. Clinician participants will receive gift cards/gifts each time they complete a survey if allowed by their health system. Alternatively, an altruistic incentive will be used where the study will contribute to a charitable organization for each returned survey.

Cost will be measured using a pragmatic method to capture all resources needed to deploy the implementation strategies [83–85]. The primary objective of the cost analysis is to estimate the cost of each strategy at the system level to gather information that will allow other decision makers to assess the resources needed to take this approach to scale within their systems. We will capture these costs by prospectively and pragmatically using spreadsheet-based templates on a monthly basis consistent with our previous studies [83, 84, 86]. These templates provide the framework for capturing costs related to each component of the implementation strategy (e.g., Epic build and maintenance; facilitation training and activities).

##### Hypotheses

We will compare the effects of two active implementation conditions, Nudge (EHR SmartList) vs. Nudge+ (EHR SmartList + facilitation) at the end of the implementation period as well as at the end of a 1-year sustainment period. We will test a total of four related hypotheses:

1. Change in the probability of target fidelity from the *pre-implementation* period to the *active implementation* period will be *equivalent* in Nudge vs. Nudge+.
2. Change in the probability of target fidelity from the *pre-implementation* period to the *active implementation* period will be *superior* in Nudge+ relative to Nudge.
3. Change in the probability of target fidelity from the *pre-implementation* period to the *sustainment* period will be *equivalent* in Nudge vs. Nudge+.
4. Change in the probability of target fidelity from the *pre-implementation* period to the *sustainment* period will be *superior* in Nudge+ relative to Nudge.

These hypotheses will also be tested with regard to the secondary implementation outcomes of reach, acceptability, and cost. Finally, we will descriptively evaluate each arm separately to determine the magnitude of change in the probability of target fidelity and other implementation outcomes over time.

#### Aim 2: Use mixed methods to identify implementation strategy mechanisms

Our understanding of the mechanisms through which the implementation strategies work is informed by previous research [73, 87] describing the practice-level mechanism, clinical adaptive reserve, through which facilitation, a practice-level implementation strategy, operates. We hypothesize that facilitation will increase practice adaptive reserve, or the ability to make and sustain change at the practice level, because it will allow for problem-solving and tailoring specific to the individual practice. Previous research [73, 87] suggests that facilitation improves practice relationship infrastructure; aligns management functions in which clinical care, practice operations, and financial functions share a consistent vision; facilitates leadership and teamwork; and improves the work environment to create a culture of learning [87]. These are all components of adaptive reserve.

##### Participants and procedure

Participants will include clinicians and health system leaders (e.g., practice directors, department chairs, and health plan directors) in the two systems. In addition to surveys assessing the hypothesized mechanism at pre-implementation and active implementation as described in Aim 1, we will also conduct qualitative interviews with a subset of clinicians (*n* = 24) and leaders (*n* = 14) at the end of the active implementation period.

##### Primary mediator

We will measure practice-level adaptive reserve using the Practice Adaptive Reserve Scale [87], a self-report practice-level measure that is completed by practice staff and aggregated into an organizational construct composed of six factors that include relationship infrastructure, facilitative leadership, sense-making, teamwork, work environment, and culture of learning. The tool has high internal consistency, has been found to be associated with greater implementation in previous cross-sectional research [88], and is sensitive to change due to facilitation [87].

##### Moderators

We will measure clinician attitudes towards firearm safety promotion in pediatric healthcare settings using questions from the American Academy of Pediatrics Periodic Survey [89, 90]. We will also examine patient demographic variables (e.g., race, ethnicity, gender identity) as potential moderators.

##### Qualitative interviews

We will conduct brief interviews with a purposive sample of clinician survey respondents (equally distributed across health system and arm) to obtain more detailed information from those demonstrating high (*n* = 12 [6 per arm]) and low (*n* = 12 [6 per arm]) fidelity measured via EHR documentation. The purpose of these interviews will be to identify additional mechanisms through which implementation strategies might operate such as motivation, self-efficacy [91], and psychological safety (i.e., safe environment for risk taking) [92]. The interview guide will be developed using the Consolidated Framework for Implementation Research [40]. We will oversample for clinicians who report firearm ownership on the survey. We will interview all leaders who agree to participate (total *N* = 20; anticipated *n* = 14). Participants will receive $25 or an equivalent gift for participation as allowed by their health system as denoted above.

#### Aim 3: Examine the effects of the adapted intervention on clinical outcomes

The objective of this exploratory aim is to examine clinical outcomes to assess the public health impact of wide-scale health system implementation.

##### Participants and procedures

As described in Aim 1, we will survey all eligible parents in the participating practices within two weeks following their child’s well-child visit.

##### Exploratory effectiveness outcomes

We will assess parent-reported firearm storage behavior, as well as youth suicide attempts, death, and unintentional firearm injuries as exploratory outcomes. Firearm storage behavior will be assessed with two questions on the parent survey that ask parents: (1) whether they have made firearms less accessible to their children since their child’s recent visit, and if so, what changes they have made, and if no, (2) whether they intend to make firearms less accessible to their children since their child’s visit. The Theory of Planned Behavior informed the development of these questions [93]. Questions were piloted with parents to ensure sensitivity and appropriateness. Responses to the intention question will be rated on a five-point Likert scale ranging from *strongly disagree* to *strongly agree.*

Youth suicide attempts, deaths, and unintentional firearm injury and mortality data will be extracted from administrative data from each health system. Relevant events will be identified via ICD-10 codes and will include all codes typically used to identify suicide attempts (including non-firearm suicide attempts) as well as official state and federal mortality records that have already been matched to health system patient records.

### Sample size calculation

Sample sizes differ by aim and approach. For quantitative outcomes, we powered on our primary implementation outcome of fidelity (i.e., parent-reported clinician delivery of the program). After accounting for non-response, we expect to include data from 18,556 parents of youth within 32 practices. Power calculations were implemented Computer Program PASS Power Analysis and Sample Size Software, (NCSS LLC, 2019) were based on a GEE test for two proportions in a cluster randomized design. Assuming an average practice size of 730 patients and an ICC of 0.03, we will have at least 89% power to detect a difference of .1 in the probability of fidelity between Nudge and Nudge+ in the active implementation period. For qualitative data, we will use purposive sampling until thematic saturation is reached (in the case of clinicians) or until all individuals within the group agree (in the case of leaders) [94].

### Data analysis

In Aim 1, the primary dependent variable is parent-reported fidelity. For each observation period (pre-implementation, active implementation, sustainment) and for each implementation condition (Nudge, Nudge+), we will describe the proportion of parents who reported having received the intervention with fidelity. We will calculate fidelity using three binary outcomes that will be modeled separately: received counseling (yes/no), offered lock (yes/no), both (yes/no). For each fidelity outcome, we will fit a single model to simultaneously examine differences between the pre-exposure and active implementation periods for both conditions as well as differences between Nudge and Nudge+. For comparing the change in the log-odds of fidelity from pre-exposure to active implementation between Nudge and Nudge+, we will use a three-sided test to simultaneously test for equivalence and superiority (as well as non-inferiority) of Nudge+ relative to Nudge [95]. Based on input from leadership in the two health systems and a review of the literature [96–98], we established that in order for Nudge+ to be considered meaningfully superior to Nudge, the difference in the change in the probability of fidelity relative to pre-implementation would need to be detect a difference of .1 in the probability. All analyses will be repeated using the sustainment period outcomes in place of the active implementation period outcomes. We will also repeat these analyses for parent-reported safe storage and exploratory effectiveness variables including youth suicide attempts, deaths, and unintentional firearm injury and mortality. Additionally, we will conduct a sensitivity analysis to explore whether intervention effectiveness varies significantly by health system.

Mediation will be tested using the product of coefficients method [99–101]. In this approach, the total effect of Nudge+ relative to Nudge will be parsed into direct and indirect effects through the mediator, practice adaptive reserve. Models will test (a) the effect of Nudge+ relative to Nudge on practice adaptive reserve and (b) the effect of practice adaptive reserve on log-odds of fidelity, controlling for Nudge+ versus Nudge. All models will include covariates to address potential mediator-outcome confounds including baseline values of the mediator and outcome variables. We will also conduct sensitivity analyses to test for an exposure-mediator interaction and will model if appropriate. An unbiased estimate of the indirect effect will be derived via the product of coefficients from the two models and confidence intervals for the indirect effect will be generated using Monte Carlo methods [100–103]. We will test the statistical significance of the indirect effect using the joint significance test [103].

Variables that potentially modify the effect of Nudge+ relative to Nudge will be tested separately by adding terms for each moderator and its interaction with the exposure to the Aim 1 models for the active implementation period. These models will estimate the conditional relationships between Nudge+ (relative to Nudge) and implementation outcomes across different values of the putative moderators.

#### Qualitative analysis and mixed methods

Text answers from open-ended survey questions with parents from Aims 1 and 3, and digitally recorded and transcribed interviews with clinicians and leaders on the mechanisms of the implementation strategies, will be loaded into NVivo qualitative data analysis software [104]. Analysis will be guided by an integrated approach [105], which outlines a rigorous, systematic method for analyzing qualitative data using an inductive and deductive process of iterative coding to identify recurrent themes, categories, and relationships. The structure of our mixed methods approach is sequential (quantitative data is primarily collected before qualitative data and quantitative data is weighed more strongly than qualitative; QUAN>qual). The function is “complementarity” (to elaborate upon the quantitative findings to understand the *how* of implementation), and the process is connecting (having the qualitative data set build upon the quantitative data set) [106]. To integrate the quantitative and qualitative results, we will follow guidelines for best practices in mixed methods [107].

## Discussion

The ASPIRE trial is a hybrid type III effectiveness-implementation trial with a longitudinal cluster randomized design. This research is a collaborative effort to combine insights from behavioral economics, diverse firearm safety stakeholders, clinicians, and health systems to test strategies to implement firearm safety as a universal suicide prevention strategy. This will be the first large-scale multi-health system study testing behavioral economic-informed implementation strategies. Both health systems included in our study indicated that they would adopt the Nudge if we can demonstrate its effectiveness in this trial, suggesting the sustainability of the proposed work. The health systems have indicated that practice facilitation (Nudge+) would need to show strong cost-effectiveness outcomes compared to Nudge for widespread adoption, considering the higher costs associated with facilitation. The evidence and insights generated can be taken to scale in the MHRN which includes 21 closely integrated health systems.

There are several strengths in the study. First, we use principles of behavioral economics and compare the effectiveness of a low cost, highly scalable implementation strategy to a more intensive strategy intended to address implementation barriers. Second, we have carefully designed our implementation strategies and adapted program based on end-user feedback, particularly firearm stakeholders who have often not been included in the conversation around firearm safety promotion in health care settings [41]. Lack of stakeholder input can be detrimental to eventual viability of programs and implementation strategies [1, 108]. Third, we will assess the costs of the implementation strategies, which have been understudied to date [109, 110]. Fourth, we will explore mechanisms of our implementation strategies; mechanistic research represents the next frontier of implementation research [48, 110]. Fifth, a strong partnership between our research team and health system stakeholders directly drives the research and we leverage the strong foundation of the MHRN. Sixth, we include an active comparison condition that is a true comparator and leverage the power of a cluster randomized trial to maximize methodological rigor.

There are also limitations. First, we did not include a control condition because our health system partners felt the public health urgency of our study topic required all practices to be assigned to an active implementation condition. Second, our reliance on EHR data to measure program reach may not sufficiently measure program delivery since it is possible for a clinician to deliver the intervention without documenting it. However, our analytic strategy to assume non-documentation reflects non-delivery of the program will, if anything, lead to conservative conclusions about program reach, and program reach may be greater than our analyses conclude. Third, our measurement for the number of cable locks taken from practices as a proxy for the maximum number of firearms that are secured after receipt of the *S.A.F.E. Firearm* program is limited, since the program could either prompt parents to secure their firearms using other locking devices that would not be captured via this metric, or could be an overestimate given that parents may take locks that they never intend to use or plan to use and don’t. Fourth, emerging evidence suggests that quick access safes are a preferred firearm storage mechanism for handguns [111], which is the most common type of firearm in the US, because they enable storage of loaded firearms for protection purposes [112]. However, quick access safes are more expensive and impractical to distribute at practices due to their size. Furthermore, cable locks work universally on nearly all types of firearms, including many handguns and long guns [113]; this contributes to their appropriateness for the present study since youth may be more likely than adults to use long guns in suicide. Offering cable locks is effective [67], and we will also distribute resources that assist parents in obtaining additional, alternative safe storage options. We will continue to work closely with lock manufacturers, potential partner organizations, and health systems to identify sustainable ways to source cable locks. Given that large health systems such as the Veterans Health Administration are moving towards purchasing and stocking free cable locks, we are confident that we will be able to work with the health systems to identify a sustainable plan for after the trial ends.

The ASPIRE trial will integrate implementation science and behavioral economic approaches to implement an evidence-based practice for firearm safety promotion in pediatric primary care. The study uses sophisticated methods to answer a question at the heart of many practice change efforts: which strategies are sufficient to support change, and why? Furthermore, the work can provide support for approaches that can bear significant outcomes for little cost. If successful, the proposed study will offer valuable insights into how best to implement evidence-based practices in pediatric primary care, particularly those that are sensitive in nature. We must act now to understand how best to implement evidence-based firearm safety programs to save the lives of American youth.

## Supplementary Information

**Additional file 1.** Standards for Reporting Implementation Studies: the StaRI checklist for completion.

## Data Availability

No study data has been collected yet. Upon study completion, any datasets used and/or analyzed during the current study will be available from the corresponding author (RSB) on reasonable request.

## Abbreviations

EHR: Electronic health record
US: United States
CFIR: Consolidated Framework for Implementation Research
S.A.F.E Firearm: Suicide and Accident Prevention through Family Education Firearm
StaRI: Standards for Reporting Implementation Studies
ASPIRE: Adolescent and child Suicide Prevention in Routine clinical Encounters
MHRN: Mental Health Research Network
HFHS: Henry Ford Health System
KPCO: Kaiser Permanente Colorado
IRB: Institutional Review Board
sIRB: Single Institutional Review Board
DSMB: Data safety and monitoring board
QUERI: Quality Enhancement Research Initiative
AHRQ: Agency for Healthcare Research and Quality
GEE: Generalized Estimating Equation
ICD-10: International Statistical Classification of Diseases and Related Health Problems – 10
